# The quantity, quality and characteristics of Aboriginal and Torres Strait Islander Australian mentoring literature: a systematic review

**DOI:** 10.1186/1471-2458-14-1263

**Published:** 2014-12-13

**Authors:** Roxanne Bainbridge, Komla Tsey, Janya McCalman, Simon Towle

**Affiliations:** The Cairns Institute, James Cook University, PO Box 6811, Cairns, 4870 Australia; Gunggandji Aboriginal Corporation, 1 Loban Lane, Yarrabah, 4871 Australia

**Keywords:** Aboriginal and Torres Strait Islanders, Evidence, Indigenous Australians, Mentoring, Social determinants of health, Systematic review

## Abstract

**Background:**

Mentoring is a key predictor of empowerment and prospectively a game changer in the quest to improve health inequities. This systematic review reports on the state of evidence on mentoring for Indigenous Australians by identifying the quantity, nature, quality and characteristics of mentoring publications.

**Methods:**

Thirteen databases were searched using specific search strings from 1983 - 2012. Grey literature was also canvassed. The resultant publications were mined to identify their outputs, nature, and quality. These were then conceptually mined for their characteristics to develop a model of mentoring that included the initiating environments, facilitating environments, operational strategies and outcomes.

**Results:**

771 citations were identified; 37 full text publications met inclusion criteria and were assessed. Fifteen were eligible for review. Four of five original research publications used strong qualitative research designs. No publications were found before 1999; the largest proportion concentrated in 2011 (n = 4). Facilitating environments included: mapping participants’ socio-cultural and economic context; formal mentoring practices with internal flexibility; voluntary participation; integrated models with wrap-around services; mentor/staff competencies; and sustained funding. Mentoring strategies comprised: holistic scaffolding approaches; respectful, trusting, one-on-one mentoring relationships; knowledgeable mentors; regular contact; longer-term relationships and exit strategies; culturally-tailored programs; personal and social development opportunities; and specialised skills and learning opportunities. Outcomes varied in accordance to program aims and included improvements in aspects of education and employment, offending behaviours, relationships, and personal, social and professional development.

**Conclusion:**

Little research explored the effectiveness of mentoring, captured its impact qualitatively or quantitatively, developed appropriate measures or assessed its cost-effectiveness. There is a real need to evaluate programs particularly in terms of outcomes and, given there were no economic evaluations, costs. Commitments to improving Indigenous Australian mentoring rely on changes to funding structures and attitudes toward research. There was insufficient evidence to confidently prescribe a best practice model. Sufficient frequency of qualitative reporting between publications concluded that mentoring is a valuable empowerment strategy in the areas of health and wellbeing, education and employment and as a remedial and preventative measure in reducing offending behaviours. An evidence-informed mentoring model would take into account the key findings of the review.

## Background

The pursuit of ways to positively influence the social determinants of health to improve health outcomes is a global mission. Viewed as a social determinant itself, empowerment^a^ and its associated strategies have become progressively evident in public health as a means of tackling the issue and equalising increasing health disparities [1]. Empowerment has application to broader populations and issues in public health, but evidence of its potential to improve a range of social determinants, health status and diverse health outcomes for socially-excluded populations is also increasing globally [1, 2]. One change strategy, mentoring, is a firm predictor of resilience and empowerment [3] and can mitigate against those circumstances that contribute to a range of underlying determinants of health inequities. Adequately implemented, mentoring assists people to build personal resilience and life competences relative to needs through strengthening support networks [4–6]. It specifically involves “the commitment of time and specific efforts by a more experienced person to the development of a mutually beneficial, supportive and nurturing relationship with a less experienced person” [7]. The landscape of mentoring literature is vast. But despite the escalating literature base on mentoring in Western contexts over recent decades [8], growth in mentoring for diverse populations is a much newer area of study, and is not nearly as abundant.

Although there has been substantial international theorisng of mentoring processes, in the Aboriginal and Torres Strait Islander Australian context, these have been piecemeal. In Australia, there is a need to recognise the distinctiveness of social, economic and political systems and unique historical circumstances of Aboriginal and Torres Strait Islander (respectfully hereafter Indigenous) Australians. Dissenting epistemologies between Indigenous and other Australians infers the likelihood of key points of difference in mentoring standards. The need to recognise differences is also supported by the fact that strategies and programs found effective in working with Western populations have not enjoyed the same success when implemented with Indigenous Australian populations [9]. It holds therefore, that in contemplating mentoring work with Indigenous Australians, acknowledging Indigeneity is a particularly important consideration because at its heart are strong values-based connections that are shared in the ensuing relationships.

Identifying the best available evidence is critical to providing a baseline for future practice, policy and research. The stronger the evidence-base that exists in a particular field, the greater likelihood that the best responses can be understood and appropriate innovations of practice can be developed and transferred in a more time-oriented manner. Conversely, progress in a field of interest can be hampered by poor quality and inaccessibility of evidence. Sanson-Fisher et al. [10] notes that the extent to which it is possible to use the best evidence to guide development in a field “will depend on the quantity and quality of available evidence”, and, that “the number of measurement, descriptive and intervention research publications across time” can indicate whether research efforts have moved beyond describing the issue to providing data about how to facilitate change. Following Sanson-Fisher et al. [10], in this article, we systematically investigated the state of the evidence of mentoring literature for Indigenous Australians with the aim of: 1) ascertaining the quantity, nature and quality of relevant published documentation across time; and 2) providing some grounding for improving the evidence-base of mentoring for Indigenous Australians by identifying the nurturing grounds for the formation of mentoring relationships and the subsequent outcomes. The purpose of the latter was to develop a general structure of Indigenous Australian mentoring that describes different forms of mentoring relationships and explains what works best for successful outcomes. The review was initially prompted by an Australian government department, who, rather than starting from scratch to continually develop and implement new local solutions to Indigenous community issues, elected to work with university researchers to understand what was already working for Indigenous people in this area. The target population was urban-dwelling Indigenous youth and families; the aim of developing and implementing the mentoring program was to improve their access to education, employment, health and housing opportunities. The overall implications of the findings for the development of mentoring initiatives for Indigenous Australians are discussed. The review has therefore gone some way to linking the characteristics of mentoring as a strategy for addressing the social determinants of health through social inclusion, engagement and empowerment and provides a significant contribution to the public health literature.

## Methods

The methodology of Sanson-Fisher et al. [10] informed the design of this systematic review. It also aligned with the approach of our five previous reviews [11–15]. We systematically appraised peer-reviewed and grey literature publications on mentoring for Indigenous Australians for the period January 1983 to October 2012; the start date coinciding with time mentoring literature entered academic writing and Kram’s [16] seminal research on mentoring.

### Aim and objectives

The overarching aim of the review was to: report on the state of evidence about mentoring initiatives for Indigenous Australian populations including children, young people and families. In the review, we critically appraised publications by:

- ▪ Taking account of the quantity of publications;
- ▪ Cataloguing publications according to nature/type;
- ▪ Mapping changes in publication outputs across the specified timeframes;
- ▪ Assessing the quality of publications; and
- ▪ Identifying the initiating environments; the facilitating environments; mentoring strategies; and mentoring outcomes.
- ▪ Taking account of the quantity of publications;
- ▪ Cataloguing publications according to nature/type;
- ▪ Mapping changes in publication outputs across the specified timeframes;
- ▪ Assessing the quality of publications; and
- ▪ Identifying the initiating environments; the facilitating environments; mentoring strategies; and mentoring outcomes.

### Search strategy

**Figure 1:**
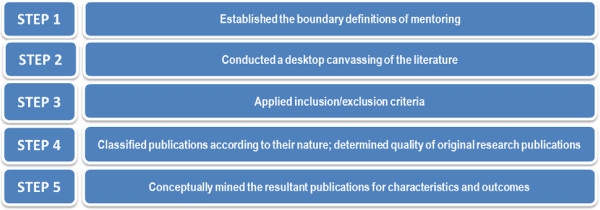
**Search strategy HERE.**

As outlined in Figure 1, a five-step systematic review method was adopted. The five-steps are described in detail below.

### Step 1: Establishing the boundary definitions of mentoring

It is difficult to gauge the effectiveness of a social innovation without defining its boundaries. For this reason, steps were taken to communicate the limits of mentoring for the purpose of this review. The boundary conditions follow the definition of mentoring articulated by Bozeman and Feeney [17] who define mentoring as: “a process for the informal transmission of knowledge, social capital and the psychosocial support perceived by the recipient as relevant…mentoring entails informal communication, usually face-to-face and during a sustained period of time, between a person who is perceived to have greater relevant knowledge, wisdom, or experience (the mentor) and a person who is perceived to have less (the protégé)”. We also took account of limitations defined by Tolan et al. [18]. The boundaries for mentoring were:

1. There was an expectation of a trusting and mutually respectful relationship developing between the mentor and protégé or mentee over an extended timeframe irrespective of whether it was formally or informally developed;
2. In the relationship, mentors held more advanced knowledge and/or experience than the mentee and were committed to providing upward mobility and support to mentees according to mentees’ needs;
3. The relationship involved the transmission of knowledge, social capital and psychosocial support according to the needs of mentees with the intention of fostering their growth and development; and
4. There was an absence of role inequality based in in the contexts of training, certification, parent-child or teacher-student relationships [18].

All publications involving Indigenous Australians - children, young people, adults and families - in mentoring initiatives were included in the search. Any mentoring innovation for any mentor or mentee was studied, regardless of the setting. We included peer mentoring, group mentoring and individual mentoring, provided the activity reported a significant mentoring component that met the study description of mentoring.

### Step 2: A desktop canvassing of the literature

A desktop analysis of literature pertaining to Indigenous Australian mentoring was conducted. We aimed to incorporate multidisciplinary literature sources in the data collection. Thirteen online databases Informit, Infotrac, Blackwell Publishing, Scopus, Web of Science, Proquest, Wiley, Taylor and Francis, JStor, the Australian Indigenous Health*Info*Net, Closing the Gap Clearinghouse, Google Scholar and Google were systematically searched by one author. Google and Google Scholar were searched to maximise search coverage of the grey literature. The first 100 returns of each, as per the Campbell Collaboration protocol for relevance and practicality (Personal Communication Campbell Collaboration, 2012) were included in the review. Reference lists of the final search documents were also scanned.

The search terms included mentor* plus Aborigin* or Torres or Indigenous. All identified publications were entered into Endnote. We searched the literature using the term mentor* only; and did not search analogous terms. Terms potentially subsumed under the rubric of mentoring, such as coaching or role-modelling for instance, were not included because of fundamental differences in their methodological approaches to the implementation of such strategies and the nature of the experiences we sought to identify. It was important to understand how mentoring is at variance with other similar interpersonal relationships and the conditions under which it is implemented and described. Haggard et al. [19] highlight the importance of this assertion: “If agreement can be reached on the fundamental, distinctive attributes that set mentoring apart from other interpersonal relationships, then researchers can incorporate boundary conditions and the issue becomes what type of mentor[ing] is being studied”. In coming to this conclusion, we examined the differences between mentoring and coaching and decided that mentoring differs from coaching in that coaching involves a more formal arrangement than mentoring with greater focus on tuition in specific experiences to achieve a defined goal in a controlled timeframe [20]. To confirm our decision, we also conducted a search for ‘coaching’ in Proquest for no returns. However, we acknowledge the potential overlapping nature of these terms and those identified in the concept of mentoring. For instance, coaching for particular a skillset is often incorporated under mentoring strategies.

### Step 3: Applying inclusion/exclusion criteria

Inclusion criteria developed at the outset of the search were applied to the retrieved documents. Publications were included if:

- ▪ The key search terms were located in the title or abstract;
- ▪ Documents were available in English;
- ▪ Documents were published between January 1983 and October 2012;
- ▪ Documents explicitly referred to experiences as ‘mentoring’;
- ▪ Documents identified Aboriginal and/or Torres Strait Islander Australians and mentoring as their key focus of concern (according to the above components of mentoring); and
- ▪ Documents compared Aboriginal and/or Torres Strait Islander Australians to other groups.

We excluded publications where the mentoring process or the effects of the mentoring innovation could not be separated from other innovations.

### Step 4: Classification of publications

The inclusion/exclusion process procured 15 final review publications. We then implemented a three-phased approach to classify their nature. In Phase 1, publications were classified according to the type of publication; in Phase 2 original research publications were classified to the domains of measurement, descriptive or intervention research; in Phase 3, inter-rater reliability was established; and in Phase 4, the quality of study publications was assessed.

Phase 1: Publications were grouped according to publication type as identified by Sanson-Fisher et al. [10]: original research, reviews, program descriptions, discussion papers/commentaries or case reports.

Phase 2: Original research publications were then classified under three categories: measurement, descriptive and intervention research:

- ▪ *Measurement research:* Publications that developed or tested a measure of mentoring for use in Indigenous Australian populations, or a measure concerned with Indigenous Australian issues;
- ▪ *Descriptive research:* Publications where the primary aim was to explore issues, processes/models or attributes related to mentoring;
- ▪ *Intervention research:* Publications in which the aim was to test the effectiveness of an intervention implemented with Indigenous Australians, such as wellbeing/empowerment, education and employment in mentoring programs. This category included research where the aim was to examine the impact of interventions designed to alter knowledge, attitudes or behaviours or to improve program delivery.

Phase 3: Thirty-seven (37/771 or 4.8%) publications were identified for full-text review. A subset of 19 publications (19/37 or 51.3% was blindly assessed by another author (a stakeholder in a mentoring program) to verify inclusion and classification of publications selected by the initial researcher. There was 79% agreement. Full consensus was then reached in negotiations between those two authors on the final decision of fifteen included publications.

Phase 4: Quality was determined using two indicators: 1) methodological quality; and 2) peer-review. Research evidence is commonly assessed by the methodological quality by which it is generated. The quality of original research publications was assessed and rated as strong, moderate or weak using the Critical Appraisal Skills Programme (CASP)’s [21] appraisal checklists for qualitative studies. Peer-review increases the probability of quality [10] and as such was used as a benchmark.

### Step 5: Conceptually mining the data

The characteristics and outcomes of mentoring were identified by conceptually mining the 15 resultant publications according to a predefined framework. This was achieved by hand-searching each publication for the framework elements. These included: author; publication year; publication type; type of program; program location; target population; numbers of participants; program aims; publication classifications; quality of the design for original research publications; initiating environments; facilitating environments; operational strategies; and mentoring outcomes. Initiating environments were defined as the boundaries within which mentoring initiatives were developed; facilitating environments, the operational elements of mentoring practice; operational strategies, the mechanisms that underlie mentoring relationships; and outcomes, those which were subsequential to Indigenous Australian experiences of mentoring.

## Results

**Figure 2:**
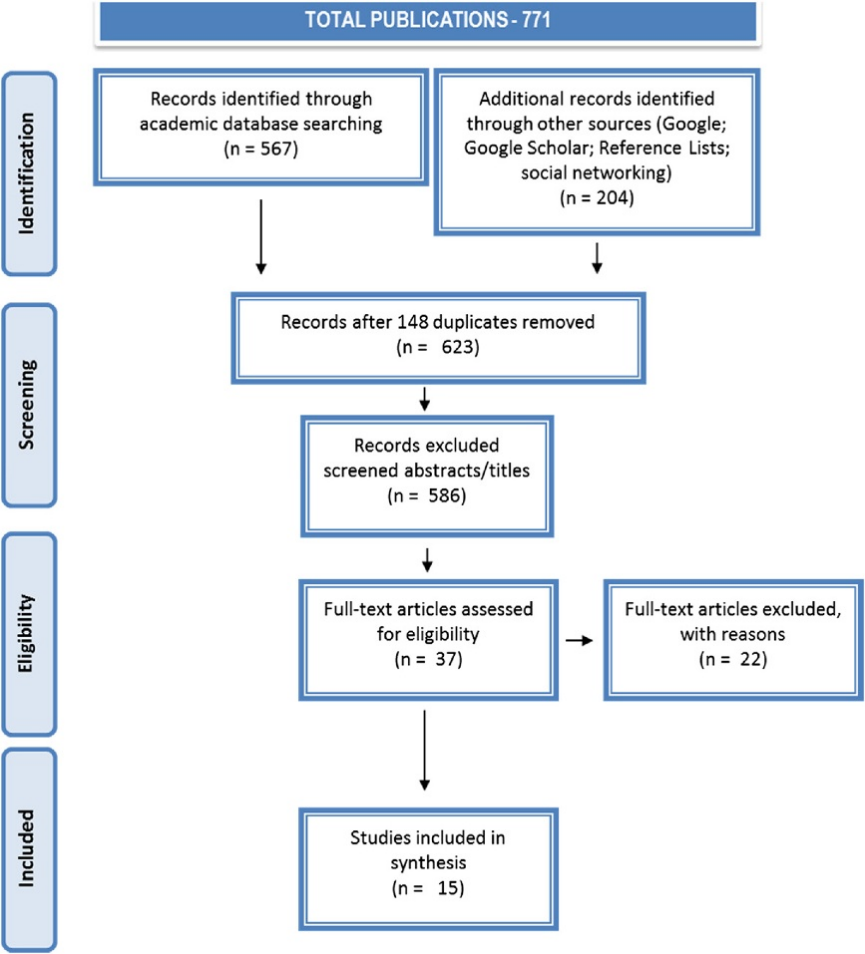
**Flow diagram indigenous Australian mentoring HERE.**

Figure 2 is a flow chart [22] of how the total number of publications identified was reduced to the final fifteen publications focussed on Indigenous Australian mentoring. In Table 1, the classifications and quality of the selected original research publications are displayed in Table 2.

**Table 1:** **Nature of classifications**

Year	All	Measurement	Descriptive	Intervention	Review	Program Description	Case Report
1999	**1**	**-**	**-**	**1**	**-**	**-**	**-**
2000	**-**	**-**	**-**	**-**	**-**	**-**	**-**
2001	**-**	**-**	**-**	**-**	**-**	**-**	**-**
2002	**-**	**-**	**-**	**-**	**-**	**-**	**-**
2003	**-**	**-**	**-**	**-**	**-**	**-**	**-**
2004	**1**	**-**	**1**	**-**	**-**	**-**	**-**
2005	**2**	**-**	**1**	**-**	**-**	**1**	**-**
2006	**-**	**-**	**-**	**-**	**-**	**-**	**-**
2007	**-**	**-**	**-**	**-**	**-**	**-**	**-**
2008	**1**	**-**	**1**	**-**	**-**	**-**	**-**
2009	**2**	**-**	**1**	**-**	**-**	**-**	**1**
2010	**3**	**-**	**-**	**-**	**1**	**1**	**1**
2011	**4**	**-**	**-**	**-**	**-**	**3**	**1**
2012	**1**	**-**	**-**	**-**	**-**	**1**	**-**
Total	**15**	**-**	**4**	**1**	**1**	**6**	**3**

**Table 2:** **The quality of indigenous Australian mentoring publications 1983 – 2012: original research publications**

Author/Publication year	Publication type	Publication classification	Quality of study design
**Burgess & Dyer, 2009**	Journal Article	Original Research	Methodological: Strong Peer-reviewed
		Descriptive	
**Nasir, 2008**	Conference Paper	Original research	Methodological: Weak
		Descriptive	
**MacCallum, Beltman & Palmer, 2005**	Conference Paper	Original Research	Methodological: Strong
		Descriptive	
**Stacey, 2004**	Report	Original Research	Methodological: Strong
		Descriptive	
**Department of Local Government, 1999**	Report	Original Research	Methodological: Strong
		Intervention Research	

### The quantity, nature and quality of identified publications

#### Number of publications

**Figure 3:**
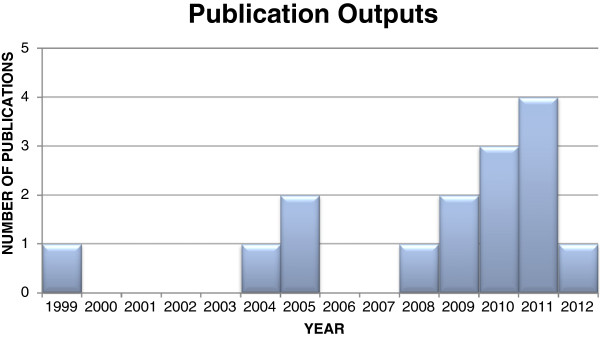
**Publication outputs over time HERE.**

A total of 771 publications were identified and included a screening of the first 100 each in Google (n = 132,000) and Google Scholar (n = 16,800). Three additional records were identified through searching references and one through a social networking site. Duplicates numbering 148 were then removed leaving 623 records. After screening titles and abstracts, 586 records were removed for not meeting the inclusion criteria; this left 37 full-text records to be more closely assessed. Fifteen of the 37 records were eligible. There were no publications from 1983 to 1998 but publications increased thereafter. The fifteen publications spanned a timeframe from 1999 to 2012; increasing from 1 per annum from 1999 to 2008, excepting 2005 (n = 2), to two in 2009, three for 2010 and four in 2011; and then to mid-2012 (at the time of the search) only 1 paper had been published (See Figure 3).

#### Classification of publications

Publications included: case reports (n = 6), journal articles (n = 4), web pages (n = 3) and conference papers (n = 2). Fourteen/15 (or 92.8%) documents were classified as descriptive. These were counted as program descriptions (n = 6) [23–28], descriptive original research papers (n = 5) [29–33], descriptive case reports (n = 3) [34–36]; and a review of mentoring programs (n = 1) [37]. Only four documents were peer-reviewed publications [23, 26, 28, 31]. Original research publications were then classified under three categories: measurement (n = 0), descriptive (n = 4) [29, 31–33].

#### Quality of publications

Because all studies were qualitative, the overall methodological quality of those publications classified as original research (n = 5) [29–33] were appraised using the CASP tool for qualitative study appraisal [21]. The overall rating of the methodological quality of original research studies was generally strong: four/five studies used strong research designs [29–31, 33]; with one rated as using a weak design [33]. Three/4 of the stronger designs were descriptive program evaluations. Methodological deficiencies of the study using the weak design included failure to adequately articulate research processes in terms of researcher relationships, ethical issues, rigorous analysis, supporting data and research value. Only one paper [29] met both quality criteria of methodological quality.

### The characteristics of mentoring

**Figure 4:**
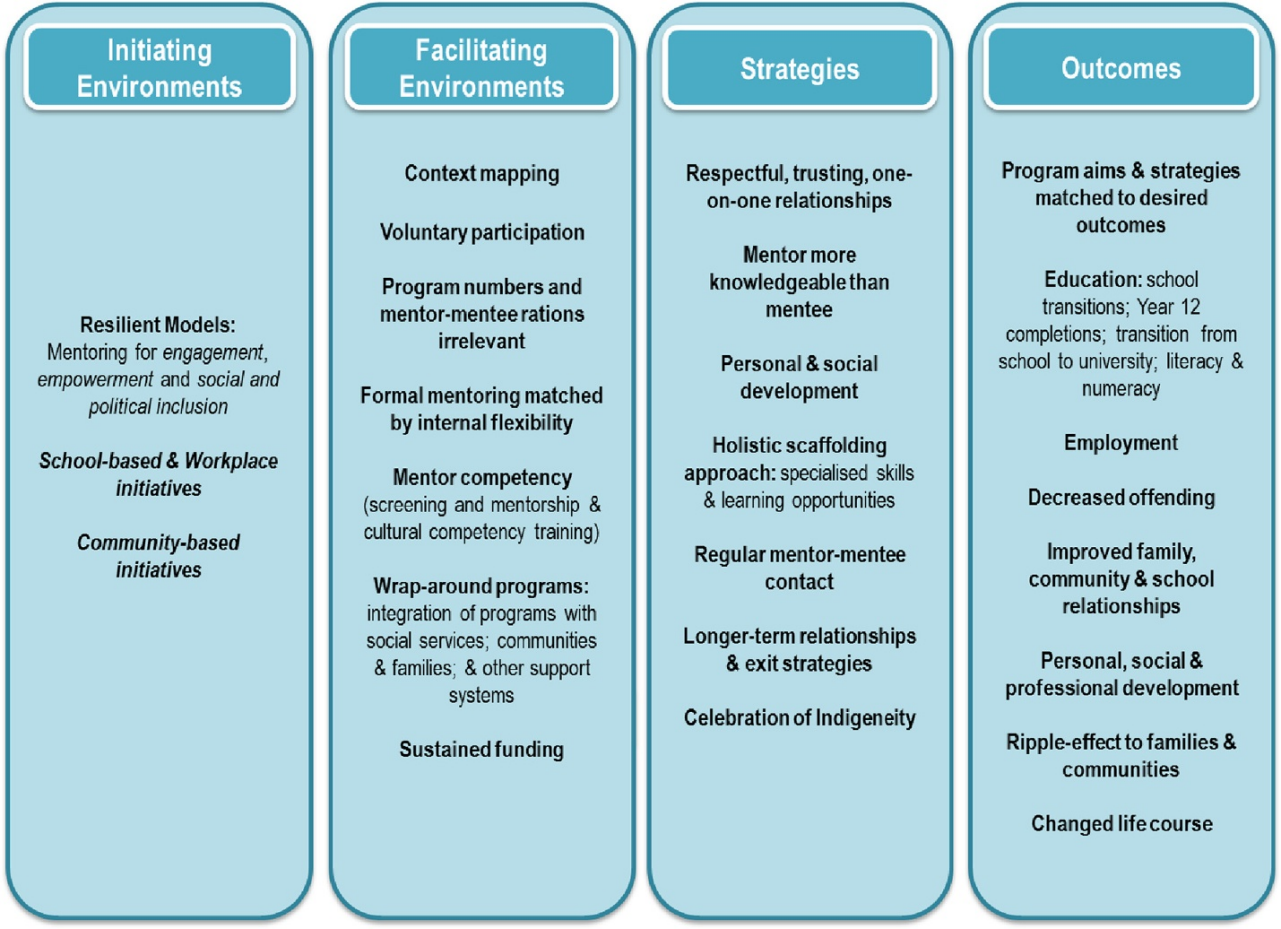
**Summary of findings: common program characteristics and outcomes.**

Table 3 displays the key characteristics of the fifteen resultant publications; while Figure 4 provides a succinct summary of the characteristics in terms of initiating environments, facilitating environments, strategies and outcomes of mentoring.

**Table 3:** **The key characteristics of Indigenous Australian mentoring publications 1983 – 2012**

Author/publication Year/publication type	Type of program	Program location	Target population	No. of participants	Program aim	Mentoring strategies	Publication classification/Quality of study design	Outcomes
**Dawes & Dawes, 2005 Journal Article**	Mentoring program for young Aboriginal and Torres Strait Islander offenders	Detention Centre	Young Aboriginal and Torres Strait Islander men	48	Successful transition of students back into their family and wider communities	Mentors trained, including cultural awareness	Program description	Positive relationships
		Far North Queensland						
						Volunteer mentors		
						Mentee choice of mentors		
					Reduce reoffending behaviour and establish links to further education and employment pathways			
						Matched on interests		
						One-on-one weekly		
						Role-modelling		
						Listening		
						Relationship building activities e.g. chess, fishing		
						Literacy, numeracy; vocationally-oriented curriculum (building and construction, art, horticulture, hospitality)		
						Links to, and support of Education Queensland		
						Funding Education Queensland and Cleveland Education & Training Centre		
**Burgess & Dyer, 2009 Journal Article**	Workplace Mentoring Program	University of Newcastle NSW	Indigenous Australian university trainees	13	Assist Indigenous job seekers enter the mainstream workforce	12 month Indigenous paid traineeship: combination of study, hands-on-learning and formal and peer mentoring	Original Research	University Certificates.
							Descriptive	12/20 completions: 8/20 completions with university employment; 4/20 completions with external university employment
							Strong	
						Trained mentors (cultural awareness)		
						One-on-one mentoring		
						Mentors matched to gender and ethnicity		
						Voluntary participation		
						Flexible to the needs of mentees		
						Partnerships with local Indigenous communities and community organisations		
						Volunteer external mentors		
						Psychological, role-modelling, counselling, acceptance/confirmation, friendship, career development		
						Funding the University of Newcastle		
**Paase & Adams, 2011 Journal Article**	Indigenous peer mentoring program	Chronic disease prevention	Indigenous people living in the inner suburbs of west Melbourne	Not reported	Developing a mentoring model to improve the health of Indigenous people	Consultation with Local Indigenous people	Program Description	Smoking cessation
								Skill acquisition
		Melbourne						
						Voluntary participation (mentees)		Increased expression of identity
						Built on existing strengths		
						Tailored to local needs and culture		Reduction in cultural isolation
						Local knowledge linked with existing services and programs		
						Partnerships with organisations		
						Group mentoring		
						Indigenous program team, peer mentors and peers locally employed		
						Formal & informal mentoring		
						Volunteer mentors		
						Trained peer mentors (as mentors and skill development); ongoing support		
						Matched cultural/socio-economic background and/or interests		
**Tribal Warriors, 2011 Journal Article**	Post-release mentoring program for young Aboriginal offenders	Redfern Community	Aboriginal young people (7-25 years) recently released from correctional centres	50 participants	Encourage education, self-respect and independence (post-release) for young Aboriginal offenders	Mentors walk with mentees through many aspects of life e.g. appointments etc.	Program Description	Developed a mentoring certificate course.Decreased re-offending including decrease of 80% in men charged with robbery
		Redfern, NSW						
						One-on-one mentoring		
						Elder leadership		
						Elder and police collaboration		
						Police-mentee and police-community liaison		
								Improved relationships between police and the Aboriginal community
						Paid mentors		
						Mentors trained, including cultural awareness		
						Training, employment and education opportunities e.g. Certified maritime training		
						Health and wellbeing training including family violence, substance abuse, fitness		
						Cultural participation		
						Funded by Department of Community Services; Department of Families, Housing, Community Services and Indigenous Affairs; Centrelink; Department of Education, Employment and Workplace Relations		
**Nasir, 2008 Conference Paper**	Mentoring program for Indigenous apprentices	Group training organisation – public sector Not reported	Indigenous apprentices	10 Indigenous apprentices, 4 field officers, 2 group training organisation managers, 3 host employer supervisors and 1 trainer	To improve the recruitment, retention and completion of apprenticeships by Indigenous Australians	Mentors recruited into the organisation	Original research	Critiqued for being ineffective
							Descriptive	
						Untrained, unsupported mentors	Weak	
						Appointed mentors		
						Mentors have dual roles in the organisation		
						One-on-one mentoring		
						Funding local training organisation		
**MacCallum, Beltman & Palmer, 2005 Conference Paper**	National Indigenous Mentoring Pilots Project	Secondary Schools; detention centres; residential schools 53 sites across Australia	Indigenous high school students	53 sites 483 mentees 332 mentors	To trial mentoring approaches to improve literacy, numeracy, attendance and participation of high school students.	One-on-one mentoring	Original Research Descriptive Strong	Mentees: Increased self-confidence and self-esteem; school attendance; retention; and participation in classroom tasks
						Trained volunteer mentors		
						1 hour per week		
						Exposure to employment pathways, community work and further education		
					To raise students’ expectations of success and the expectations of their parents and teachers			
								Enhanced valuing of school and connections between school and work
						Awards Celebrations		
						Life skills		
						Role-modelling		
						Identity building		Increased ability to solve personal and social problems
						Motivational speakers		
						Social activities- fishing, gardening, dance, art, sport, camps		Development of leadership and life skills
						Reconciliatory approach		
								Improved relationships with, and between peers, teachers and family members
						Vocational educational experiences		
						Recognition of Indigeneity		
								Improved literacy and numeracy
						Respectful relationships		
						Mentor humour		Mentors: improved knowledge of Indigenous culture and youth issues; development of strong relationships with students; enhanced personal development and self-esteem.
						Involvement of families and communities		
						Genealogy program		
						Networking for employment		
						Funding Department of Education, Science & Training		
								School and Community: enhanced links between school and community; increased involved of families in school; awareness of, and access to local Indigenous role-models; development of inter-school relationships; positive contact between Indigenous and non-Indigenous families.
**Stacey, 2004 Report**	Panyappi Indigenous youth mentoring service	Indigenous Youth Mentoring Program South Australia	Indigenous youth ‘at-risk’ at of being a victim of crime or engaging in offending behaviour.	30 Urban (inner city) Indigenous youth 10-17 years	To intervene in pathways of offending behaviour	Works from a development perspective	Original Research	Marked change in offending behaviour
						Family-inclusive approach	Descriptive	
					To decrease each young participant’s contact with the juvenile justice system and/or agencies associated with this system.			Attitude shift
						Accredited trained paid mentors including cultural awareness		
							Strong	
								Decreased frequency of offending
						Mentor support and supervision		
								Increased self-belief, and personal and cultural identity
					To promote self-discovery and self-determination by young people participating in the program their family and wider community	Formal longer-term mentoring		
						Referrals but voluntary		
						participation		Reduced stress
						Cultural fit		Decreased contact with the juvenile justice system
						Developing a positive, caring and non-		
						judgemental relationship		Reduced formal cautions, court orders, family conferences and convictions
						One-on-one intensive support – 15-20 hrs/wk		
						Building networks of support around the individual – schools, youth health, welfare		
								Services enabled to work better with young people and their families
						Support of external agencies		
						Group strategies		
						Mentoring beyond the trouble period		
						Role-modelling		
						Accompanying mentees to appointments		
						Access to education, training and recreation		
						Genealogy program		
						Mentoring timeframe 2-17 months		
						Art program		
						Encouraging relationships with family, parents and community		
						Enabling opportunities to experience success		
						Tutoring		
						Life skills		
						Providing a safe environment		
						Funding Attorney Generals Department		
**Brereton &Taufatofua, 2010 Report**	Indigenous Australian Mentoring Programs in employment	Workplaces Australia-wide	Indigenous Australians	Not relevant	Overview of Indigenous mentoring programs across Australia aimed at increasing participation in employment	Tailored to individual needs	Review Descriptive	Only ‘expected’ outcomes reported
						Flexibility of the workplace		
						Resources, training and support for the mentor and mentee		
						Cultural awareness training for Indigenous and non-Indigenous mentors		
						Acknowledging difference		
						Incorporating and valuing local knowledge		
						Experiential learning		
						Formal evaluation for program improvement		
						Role-modelling		
						Links to support resources and services		
						Appropriate empathy and listening skills		
						Accountability and reliability of mentors and mentees		
						Developing rapport and building trust		
						Setting and reaching goals for the mentee		
						Bridging closure to the relationship		
						Funding not relevant		
**Australian Indigenous Mentoring Experience, 2009 Report**	Youth Mentoring into Education	Australian secondary schools	Indigenous secondary school students Years 9-12	4 universities; 13 staff; 325 mentees; 500 mentors; 30 high schools	Increase Year 10 & 12 progression rates	One-on-one mentoring	Case Report	Increases across progressions for Years 9-10 (88% AIME compared to 81% National); Years 10-11 (81% AIME compared to 59% National); Years 11-12 (92% AIME compared to 63% National)
							Descriptive	
					Increase Year 12 to university progression	Voluntary participation		
		East Coast of Australia						
						Voluntary mentors		
					Work with 6000 Indigenous secondary school students by 2020			
						1 hour/wk for17 week intensive program		
						Learning Centres		
						Community & University Engagement		
								Increases Year 12 completion (73% AIME compared 60% National)
						Role-models		
						Shared social activities		
								Increases Year 12 to university (38% AIME compared to 1.25% National)
						Funding partners (Universities), philanthropic organisations, in-kind support, fund-raising		
						Linking in mentees family and community		
**Australian Indigenous Mentoring Experience, 2010 Report**	Youth Mentoring into Education	Australian secondary schools East Coast of Australia	Indigenous secondary school students Years 9-12	7 universities; 388 mentees	Increase Year 10 & 12 progression rates	One-on-one mentoring	Case Report Descriptive	Increases across progressions for Years 9-10 (88% AIME compared to 91.5% National); Years 10-11 (87% AIME compared to 74% National); Years 11-12 (86% AIME compared to
					Increase Year 12 to university progression			
					Work with 6000 Indigenous secondary school students by 2020			
						Voluntary participation		
						Voluntary mentors		
						1 hour/wk for17 week		
						intensive program		
						Learning Centres		
								66.7% National) Increases Year 12 completion (100% AIME compared 71.8% National)
						Community & University Engagement		
						Role-models		
						Shared social activities		Increases Year 12 to university (38% AIME compared to 10% National)
						Funding partners (Universities), philanthropic organisations, in-kind support, fund-raising		
**Australian Indigenous Mentoring Experience, 2011 Report**	Youth Mentoring into Education	Australian secondary schools	Indigenous secondary school students Years 9-12	10 universities;30 staff; 566 mentees	Increase Year 10 & 12 progression rates	One-on-one mentoring	Case Report Descriptive	
						Voluntary participation		
					Increase Year 12 to university progression	Voluntary mentors		
		East Coast of Australia						
					Work with 6000 Indigenous secondary school students by 2020	1 hour/wk for17 week intensive program		
						Learning Centres		
						Community & University Engagement		
						Role-models		
						Shared social activities		
						Funding partners (Universities), philanthropic organisations, in-kind support, fund-raising		
**Department of Local Government, 1999 Report**	Aboriginal Political Mentoring Program	Local Councils	Local Aboriginal community members	27 participants	Encourage Aboriginal people to run for local elections	Voluntary participation by mentees but selection process	Original Research	Not able to be definitively assessed: Increased political participation - 11/27 ran for election and 2 were elected
		Kyogle Area NSW						
							Intervention Research	
					Educate the Aboriginal community on the local government process and the importance of Aboriginal input into local government			
							Strong	
						Mentors - existing relationships with mentee and Aboriginal community; understanding of Aboriginal culture; vast experience of local government and supervision		Increases across progressions for Years 9-10 (97% AIME compared to 91.5% National); Years 10-11 (92.6% AIME compared to 74% National); Years 11-12 (79% AIME compared to 66.7% National)
						Mutual matching		
						One-on-one mentoring but multiple mentors		
								Increases Year 12 completion (87.5% AIME compared 71.8% National)
						Supported in political activities and social support		Increases Year 12 to university (35.7% AIME compared to 10% National)
						6 months + relationship		
						Funding Department of Local Government NSW		
**PASS Australia 2012 Web Page**	Mentoring Program for Indigenous students in years 10, 11 and 12	Secondary Schools Queensland	Indigenous secondary school students	525 students in 2011	To improve the lives of Indigenous Youth, through Education, Leadership and Mentoring, by providing them with the tools to become strong community leaders for the future	Group and one-on-one mentoring	Program Description	Program graduations
						Voluntary participation (mentees) Paid mentors		Vocational qualification - TAFE Certificates
						1 day per week for 2 years		
						Indigenous and non-Indigenous mentors		
						Paid employment		
						Exposure to employment pathways, community work and further education		
						6-9 Merit points towards School Certificate		
						Cultural program		
						Sports program		
						Career Pathways Program		
						Leadership		
						Education to employment transitioning		
						Industry work experience		
						Industry, community and school partners		
						Awards Celebrations		
						Funding Department of Education, Employment & Workplace Relations		
**New South Wales Government, 2010 Web Page**	Workplace Mentoring Program for Aboriginal people working in the NSW public sector	Public Sector NSW	Aboriginal people working in the NSW public sector	Not relevant	Mentoring guidelines to improve employment, training and career development opportunities for Aboriginal people in the NSW public sector	Modelling desirable behaviours and attitudes	Program Description	Not relevant
						One-on-one mentoring		
						Helping the mentee understand the values of the agency		
						Actively listening to the mentee		
						Sharing your own relevant stories and experiences		
						Helping the mentee to identify their goals		
						Providing opportunities for learning and reflection		
						Understanding of cultural obligations		
						Encouraging the mentee to develop new skills		
						Offering career advice		
						Guiding the mentee to achieve objectives		
						Providing insights into the culture of the agency		
						Offering constructive feedback; and providing regular encouragement		
						Funding NSW Government		
**Show Me The Way Mentoring 2011 Web Page**	Online mentoring program for Indigenous students	Schools NSW	Young Indigenous students in school or in school-based traineeships	14 students in 2010	To encourage Indigenous students to stay at school and go on to tertiary education.	Program matched to government policy	Program Description	New program - not reported
				29 school-based trainees 2011		Technology-driven mentoring strategies – online face-to-face mentoring contact; development of career videos		
					To self-empower Indigenous students to understand what's involved in developing a career path in conjunction with learning/mentoring partners with real world experience.			
						Mentor training including cultural training		
						Voluntary participation (mentors/mentees)		
						Mentor competency tested		
						30 mins/fortnight minimum participation		
						Video workshops (careers and trades)		
						One-on-one mentoring		
						Role-modelling		
						Matched mentoring to student needs		
						Tailored to Indigenous youth		
						Flexibility that meets the needs of individual students		
						Minimal in-person face-to-face meeting		
						Face-to-face literacy, numeracy and media literacy approaches; and training for students, teachers and corporate learning partners		
						Professional program development		
						Participation at school and home		
						Partnerships students, school, staff and community		
						Laptops supplied to students for access and engagement		
						Implemented discretely, and in conjunction with existing school programs		
						Continuous quality improvement strategies embedded in the program		
						Reward system for participation		
						Funding – charitable organisation and corporate partners

#### Initiating environments

The fifteen eligible publications focussed on Indigenous Australian mentoring implemented in various places across Australia. Mentoring approaches encompassed a range of program designs that addressed specific issues and subsets of the Indigenous Australian population. They were spread across several different contexts: workplace [n = 4], at-risk youth [n = 3], secondary education [n = 6], health promotion [n = 1] and political participation [n = 1]. Nine (60%) publications were concentrated on youth mentoring.

Most publications were centred on mentoring programs that aimed to improve education and employment outcomes for Indigenous Australians (11/15 or 80%). This focus was in sharp contrast to publications (3/15 or 20%) described as program innovations to address specific risk areas or problem behaviours, for example, changing offending behaviours for ‘at-risk’ youth [33]; young offenders [23]; and post-release young people [28]. Programs with educational and employment goals accounted for 11/15 (73.3%) publications: there were six (40%) secondary school-based mentoring programs documented and five (33%) workplace initiatives. School-based programs expanded rapidly over the past seven years, from one in 2005, to five being developed from 2009 to 2012. Site-based programs in the workplace were initiated in 1999; the other four more recent publications were spread from 2008 to 2010. Thus academic and employment-related aims were driving most of the programs. Only three/15 publications [28, 31, 33] set attitude change as part of their explicit aims. The first sought change in the expectations of success for Indigenous Australian students for students, families and teachers; and the second, to promote self-discovery and self-determination; and the last sought to encourage self-respect and independence.

### Facilitating environments

The facilitating environments, in which mentoring relationships developed, incorporated the operational elements of mentoring practice. Varied participant numbers were found across publications reporting figures in mentoring programs. They ranged from smaller programs with 10 mentees [32], 13 mentees [29] and 14 mentees [27] to 566 mentees [36]. The largest numbers were evidenced in three school-based programs: PASS Australia [26] had 525 mentees; AIME [34–36] had 483 mentees, 366 and 566 respectively; and there were 483 mentees in the National Indigenous Mentoring Pilots Program [31]. In all cases these participant numbers were distributed across a number of sites, thus reducing the number of participants at each site significantly. For example, 483 mentees in the National Indigenous Mentoring Pilots Program was spread across 53 sites; therefore averaging 9 mentees per site.

All publications reported planned and systematic organisation of mentoring practices; nevertheless, in 9/15 or 60% publications voluntarily participation of mentees in programs were reported; the nature of participation for mentees was not mentioned in all other cases. The skills, qualities and competencies required by mentors to fulfil their commitments to Indigenous Australian mentees were taken into account. Eight/15 (53.3%) publications described having mentor training [23, 26–29, 31, 33, 37]; seven of these included cultural awareness training as one aspect of their training. The Tribal Warriors Program [28] developed locally-tailored mentoring accreditation as part of their program. Another, Nasir [32] critiqued the incidence of untrained mentors. In all cases, the mentor was considerably more knowledgeable than the mentee.

The matching of mentors to mentees either in terms of mutual connections [30], gender and ethnicity [29]; socio-economic and cultural background [25] and/or areas of interest/expertise [23, 25] according to mentee needs [25, 27] was evidenced in five cases. In one instance, mentees had a choice of mentors [23]. In other cases, mentors were either not matched to mentees, or this was not reported. While many publications [23, 25, 27, 29, 31, 34–36], 8/15 or 53.3%, stated that mentors volunteered, 4/15 or 26.6% publications [26, 28, 32, 33] reported employing paid mentors in their programs; and three cases [24, 30, 37] did not report on this aspect of mentoring. The value of supporting the welfare of mentors registered in only 2/15 (13.3%) publications; a lack of support for mentors was however, critiqued by Nasir [32].

Five/15 (33.3%) publications explicitly demonstrated a reconciliatory approach other than partaking in cultural awareness training. For example, in the case of implementing a health promotion program, Paase and Adams [25] clearly embedded Indigenous protocols in their ways of working in the development of a peer mentoring program. This was evidenced by their reported consultations, valuing of local knowledge, cultural tailoring and delivery of the program. A high premium was also placed on the integration of mentoring with social services, communities and families and other support systems such as welfare agencies, corporate and industry partners, police, schools, health services, universities and training and recreation services; this was evidenced in 13/15 (%) publications [23, 25–29, 31, 33–36].

Resourcing for mentoring programs reported (13/15 or 86.6%) was weighted in favour of government-derived funding with 7/13 or 53.8% [23, 24, 26, 28, 30, 31, 33, 37] identifying various government departments as their supporting body. Other funding sources ranged from charitable organisations that had the support of corporate partners [27] and programs which were dependent on partner organisations, philanthropic investments, fund-raising and in-kind support [34–36] to workplace backing [29, 32].

### Strategies

Mentoring strategies are the mechanisms that underlie mentoring relationships. While publications contributing to the review described diverse and sometimes poorly delineated processes in mentoring programs, four common program elements were evident in the mentoring models. These strategies were: 1) developing respectful, trusting, one-on-one mentoring relationships; 2) promoting personal and social development; 3) integrating social services, organisations and other support systems including communities and families; and 4) increasing specialised skills.

*Developing respectful, trusting, one-on-one mentoring relationships* was reported in 13/15 or 86.6% of cases [23, 25–29, 31–37]. In the two other cases, PASS Australia [26] used group and one-on-one mentoring, while the health promotion program [25] used peer group and one-on-one mentoring practices. Seven/15 publications reported timeframes: School programs AIME [34–36] and National Indigenous Mentoring Pilots Program [31] invested one hour per week; Show Me The Way [27] a minimum of 30 minutes fortnightly; PASS Australia [26] one day per week; while the Panyappi Program [33] reported an intensive 15-20 hours weekly with young people. Strategies used to create trusting relationships between mentors and mentees included aiming for longer-term relationships; social/shared activities such as fishing, gardening, dance, sports, art, chess and camps and group activities; strategies that promoted friendships such as mentors sharing personal stories, being reliable and accountable, and providing constructive feedback and regular encouragement; demonstrated acceptance of mentees including empathy, non-judgemental attitudes, caring, humour, listening and bridging closure to relationships; mentors walking through life with mentees; and cultural, gender and interest matching.

*Promoting personal and social development* featured as a common and necessarily important strategy occurring in programs and mentor-mentee interactions. This saturation occurred with the exception of Nasir [32]. Nasir criticised mentoring practices in an organisation that aimed to improve the recruitment, retention and completion rates of Indigenous Australian apprentices. She argued that mentoring in this instance was one-way and neglected to take account of Indigenous Australian ways of knowing, being and doing in practice. Fourteen/15 or 93.3% of publications explicitly incorporated acknowledging Indigeneity as a strategy for promoting personal and social development for mentees. There was acknowledgement of the centrality of cultural mores for Indigenous Australian people to attend to the complexity of cross-cultural situations and acknowledgement of historical power relations. Recognised across publications was that when groups of Indigenous Australians gather together, there is important cultural work conducted in terms of sharing and making meaning of Indigeneity. Driving strategies that acknowledged Indigeneity were recognising Indigenous ways of knowing, being and doing by having the consistent endorsement of, and valuing of Elder/family/community-inclusive relationships and knowledge. Programs were thus culturally-tailored to mentee needs. Various other approaches used to translate this strategy into practice ranged from having Indigenous Australian reference groups guiding mentoring processes, through having the flexibility in program implementation to account for cultural protocols and differences, incorporating genealogy programs to connect with identity building work, and partnering with local Indigenous Australian organisations, to engaging Indigenous Australian role-models as the faces of programs.

Most publications, 12/15 (80%) described *integrated mentoring models* - implementing a circle of care and support around mentees to support personal and social development. The three cases in which outside support was not integrated were all workplace mentoring programs [24, 30, 32]: Nasir [32] critiqued the program supporting apprentices for this absence. This holistic scaffolding approach meant that programs built-in networks of support and resources around mentees as part of their mentoring practices. Stacey [33] demonstrated this well in her evaluation of Panyappi, a youth mentoring program. It aimed first to intervene in pathways of offending behaviour and second, to decrease young participants’ contact with the juvenile justice system and/or agencies associated with this system. A third aim sought to promote self-discovery and self-determination by young people participating in the program, their families and wider community. Stacey reported that Panyappi worked in conjunction with a range of health, family, education and community services. The integrated model also had mutual benefits for the program and services; it enabled services to work better with young people and their families and referrals were received from these services to Panyappi. This was also an outcome of the mentoring relationship. In addition to the previously-mentioned support structures, other wrap-around services in integrated models included psychological support and counselling. These strategies served to strengthen social and cultural capital; this allegedly worked both ways in mentoring relationships – for mentees and mentors.

All publications focussed to varying degrees on *increasing specialised skills* for mentees, and to a lesser extent building mentor skills. Strategies varied according to the nature of the mentoring program (its goals, objectives and the backgrounds of mentees and mentors) and the existing skill sets within that cohort. Matching appropriate skills and development to the program aims were crucial for the overall success of programs and was resourced as much as possible through the mentors. For example, the AIME program [34–36] had academic achievement and university enrolment as goals and thus situated the program in schools, incorporated Learning Centres into their program and co-ordinated with local universities to recruit their mentors.

*Increasing specialised skills* for mentees and mentors also involved accessing the cadre of organisations, services and/or other human resources that were integrated into the programs to support skill development. Targeted skills sets for mentees focussed on learning opportunities including literacy and numeracy; career development skills such as resume writing and work ethic and work experience; areas in the achievement of mentee goals such as education in the political system; and vocationally-oriented curriculum (building and construction, art, horticulture, hospitality and maritime). For mentors, increased skills most often took the form of cultural competency training and professional development through mentoring courses.

### Outcomes

Not all publications recounted the extent to which programs were effective. Of the nine that did, the rationales for program development intimated that a range of outcomes was being targeted. To a large extent, outcomes were matched to program aims. At times however, consequences of programs were unexpected. Most outcomes were qualitatively investigated and described, with only three reports quantitatively measuring their achievements [34–36]. AIME provides an excellent example of the aforementioned. The goals of the AIME Program for 2009, 2010, 2011 were to work with Indigenous Australian secondary students to improve student progressions between Years 9, 10, 11 and 12; increase the number of Year 12 completions; and grow the numbers of students transitioning from Year 12 to university. Thus measures reflected these goals and showed significant changes. In 2009, 2010 and 2011 respectively, and corresponding to AIME’s targets, reported were increases across progressions for Years 9-10 (88%, 88%, 97% AIME compared to 81%, 91.5%, 91.5% Indigenous students Nationally); Years 10-11 (81%, 87%, 92.6% AIME compared to 59%, 74%, 74% of Indigenous students Nationally); and Years 11-12 (92%, 86%, 79% AIME compared to 63%, 66.7%, 66.7% Indigenous students Nationally). There was also increases for Year 12 completions (73%, 100%, 87.5% AIME compared 60%, 71.8%, 71.8% Indigenous students Nationally); and increases for Year 12 to university (38%, 38%, 35.7% AIME compared to 1.25%, 10%, 10% Indigenous students Nationally).

Likewise the Tribal Warriors program [28] for post-release Indigenous Australian men described decreased re-offending, including a decrease of 80% in men charged with robbery; and improved relationships between police and the Aboriginal community. Dawes & Dawes [23] also commented on positive relationships as the outcome of their program for young people in a detention centre. In the Panyappi mentoring program for young people who had, or were at risk of offending behaviours, Stacey [33] qualitatively reported: marked changes in offending behaviour; decreased frequency of offending; decreased contact with the juvenile justice system; and reduced formal cautions, court orders, family conferences and convictions. Other outcomes that corresponded with program aims included attitude shifts; increased self-belief, and personal and cultural identity; reduced stress; and enhanced capacity of services to work better with young people and their families.

In school environments, MacCallum, Beltman & Palmer (2005) [31] found that mentees experienced increased self-confidence and self-esteem; school attendance; retention; and participation in classroom tasks; enhanced valuing of school and connections between school and work; increased ability to solve personal and social problems; development of leadership and life skills; improved relationships with, and between peers, teachers and family members; and improved literacy and numeracy. They also revealed that mentors showed improved knowledge of Indigenous Australian culture and youth issues; development of strong relationships with students; and enhanced personal development and self-esteem. The flow-on effects of the mentoring program impacted beyond the central mentoring relationships to the school and community: enhanced links between school and community; increased involved of families in school; awareness of, and access to local Indigenous role-models; development of inter-school relationships; and positive contact between Indigenous and non-Indigenous Australian families. Similarly, in the university setting, Burgess and Dyer [29] showed that of 12/20 program completions, 8 gained internal university employment; and four found external university positions. The health promotion mentoring program [25] identified smoking cessation, skill acquisition, increased expression of identity, and a reduction in cultural isolation as program outcomes.

## Discussion

### Quantity, nature and quality

The findings show that literature around Indigenous Australian mentoring has an emergent lifespan of 14 years. They revealed that while outputs have increased, the volume of original research publications has not increased across time. All original research outputs were qualitatively-oriented. The methodological adequacy of the identified original research publications was strong; however, only one was peer-reviewed. There was no pattern in the volume of original research publications; these were randomly dispersed in number and nature across a 10 year lifespan from 1999 – 2009. This is not surprising given that publication outputs were heavily weighted in favour of descriptive documents: fourteen/15 publications were labelled descriptive and only one as intervention research. Specifically, such an output record means that Indigenous Australian mentoring research is still in its early exploratory phase and little research has explored the effectiveness of mentoring in its various forms, captured its impact qualitatively or quantitatively, developed appropriate measures or assessed it cost-effectiveness. This pattern is consistent with the results of several other reviews across a number of Indigenous-specific areas, including health, program transfer and implementation, sexual assault, cultural competency, child and maternal health and suicide prevention [10–15]. The stop-start nature of Indigenous funding patterns, lacking consideration for the inclusion of evaluative components in program implementation and short-term research funding all slow contributions toward the productive trend of more rigorous strategic intervention research [10, 38]. While qualitative research is valuable in setting a baseline, it might “not maximise research benefits” [10]. Thus more compelling advances in the field of mentoring require paradigm-shifting approaches. Despite the concentration of included publications on describing the elements and outcomes of Indigenous Australian mentoring experiences^b^ however, these can provide valuable evidence and lessons for developing the field further. The slow progression of Indigenous Australian mentoring research is consistent with the findings of others in the broader field of mentoring. For instance, Crisp & Cruz [39] noted that “mentoring research has made little progress in identifying and implementing a consistent definition and conceptualization of mentoring, is largely atheoretical and is lacking in terms of rigorous quantitative research designs that allow for testing the external validity of findings”.

### Mentoring characteristics

The main characteristics of Indigenous Australian mentoring were summarised in Figure 4. The key components of Indigenous Australian mentoring aligned quite closely with non-Indigenous and international theorising on mentoring. These theorists suggest that mentoring work, as also evidenced in this review, must fit the needs and characteristics of the target group. Ideally, program elements should work in synergy, but those deemed most important and supported by this review are: duration (length of the relationship); intensity (frequency of contact); extent of integration with other services; programs matched to target groups (high-risk populations vis-à-vis high-performance populations); a focus on approaches (formal or informal and cultural appropriateness) [7, 40–43]; sustainable funding arrangements; and community buy-in [44]. The key differences found in this review were the ways programs accounted for and celebrated cultural differences in their implementation. This difference meant strategies such as training in cultural competencies became important; along with screening mentors for individual characteristics and attitudes such as respect and acceptance. However, to be noted is that there is limited evidence to suggest a lack of fitness between overseas mentoring models and local contexts [5]. In fact, DuBois et al. [4] stated that the practice of matching on race, gender and interests does not impact program effectiveness to the degree initially thought.

This review included a range of mentoring initiatives aimed at increasing Indigenous Australian empowerment, engagement and social inclusion across a number of different developmental areas; particularly in education (school-based initiatives), employment (workplace initiatives) and broader health and wellbeing. Initiatives were embedded in ecological models of resilience models that worked from the premise of providing opportunities for building on existing strengths for mentees, mentors, families and communities, and service providers. In individual programs, mentee numbers and mentor-mentee ratios varied and appeared irrelevant; that is, outcomes were equally recorded against programs regardless of the participant intake or mentees allocated to each mentor. All publications predominately described a clear orientation toward formal mentoring processes, meaning that while participation for mentors and mentees was voluntary and there was internal flexibility, there were certain non-negotiable aspects and formal organisation of programs. Generally however, there were a number of flexible elements that contributed to their success. Thus developed were innovative integrated models of mentoring programs with wrap-around social services, communities and families and other support systems. These models had a variety of educational and life skill-related supports and in some instances clinical support e.g. psychologists. Consequently, these models link mentees and mentors, and also connect many individuals and resources in the mentors’ lives to the networks in the lives of the mentees. Since both mentees and mentors are connected to the program staff and service providers this approach has important implications for providing circles of holistic care needed to help programs deliver more targeted mentoring. To engage in this type of social mapping, it was evidenced that mentoring programs need to have a clear understanding of the environment and socio-cultural and economic milieu in which potential participants operate as well as clearly defined aims and outcomes to make a good match with strategies. How these criteria manifest, informs the type of program and its operational strategies.

In order to enhance the benefits of mentoring, a focus on mentees’ support and development was matched with training and development of mentors. Mentor competency, particularly training in mentorship and cultural awareness were evidenced as critical prerequisites for successful program implementation. Given the long-term nature of successful mentoring practices, sustained funding was critically noted as vital to maximising the benefits of mentoring.

Mentoring strategies were manifest in many different forms. As previously noted, mentoring was often reported as one strategy of a more extensive support/development program. Programs were culturally-tailored to meet mentee needs, both through implementing the integrated model approach (inclusion of Elders, families and communities) and acknowledging Indigeneity by ensuring cross-cultural training was provided to mentors. But support for Indigenous Australians through mentoring, like most other Indigenous programs, has often used short-term approaches. This approach is contrary to the evidence found in this review that longer-term investment is needed. The call for longer-term mentoring is made in international mentoring literature. Indeed, this evidence that suggests inadequate implementation and short-term programs has potentially harmful effects on mentees [6, 45]. This review showed that mentoring impacts most positively within a respectful longer-term mentoring relationship characterised by trust and regular one-on-one mentor contact. The amount of contact time in the mentoring relationship was flexible; however, the more vulnerable the mentee the more contact time needed was reported. Strategies also featured opportunities for reciprocity between mentees and mentors. However, mentors were considered to be more knowledgeable, particularly in the field in which the program developed. One important but not necessarily intended outcome of such engagement was that whether or not programs committed to a reconciliatory approach, respect for Indigenous Australian cultures and reconciliatory attitudes were consequences of mentoring processes in a number of cases.

Personal and social development of the mentee was supported by a holistic scaffolding approach in which specialised skills and learning opportunities were provided. These development opportunities also extended to mentors. Mentors were provided with training in mentorship to ensure they had knowledge of and exhibited the most engaging mentor characteristics.

The ways mentoring was used to support Indigenous Australians looked very different across program aims, and therefore a diverse number of outcomes were cited. Significant outcomes were realised in the largest proportion of mentoring publications, with clear benefits to both mentees and mentors, and others: with the exception of AIME reports [34–36], these benefits were not quantified. The nature of outcomes for Indigenous Australians however, matched the wide range of positive outcomes that are evidenced in other mentoring programs; for instance, behavioural, attitudinal, health including social and emotional wellbeing, interpersonal, motivational, and academic outcomes [4–6]. According to the broader non-Indigenous literature, mentoring benefits are more apparent for those facing complex environmental risks [42]; thus mentoring represents a good strategic investment for Indigenous people who have diverse needs. Because the mentoring process involves a reciprocal relationship in which all parties assume active roles, it needs to be identified as an investment in community development as well as the individual development of mentees.

### Limitations

A rigorous search strategy was used and care taken to canvass a broad spectrum of multi-disciplinary peer-reviewed and grey literature. However, there are always possibilities of oversight when searching publications and thus potential that all relevant studies were not located. For instance, the omission of the analogous search terms might have meant that we missed relevant mentoring studies subsumed under different terminology; and search facilities in individual databases differed. Relevant publications might have been misclassified or excluded. Nevertheless, elevated levels of agreement between blinded coders suggest relevant publications have been classified or excluded with high level accuracy. Timeframes canvassed began from the emergence of the earliest mentoring literature and thus allowed for the optimal extraction of patterns of publication quantities and methodological changes. A careful hand-searching of included publications was implemented to mine for relevant data on mentoring characteristics. However, the human component of implementation means it is possible some elements were not located.

### Conclusion

The primary outcome of the review indicates that there is currently insufficient evidence from published documents to confidently prescribe a best practice model for Indigenous Australian mentoring. Nevertheless, there was sufficient frequency of qualitative reporting between the authors of publications to conclude that mentoring is potentially a valuable empowerment strategy, particularly in the areas of health and wellbeing, education and employment and as a remedial and preventative measure in reducing offending behaviours.

There is a lack of evidence from peer-reviewed publications or in the grey literature on the most effective strategies for mentoring Indigenous Australians. The only measurable outcomes for effective mentoring practices lies in mentoring programs for Indigenous Australian secondary students [34–36]. Importantly, the review of mentoring programs shows that there are different forms of mentoring that offer considerable promise for increasing participation rates in education and employment and in improving wellbeing (including health benefits, more active citizenry and socio-cultural inclusion and decreased risk of offending) for Indigenous Australians. An evidence-informed mentoring model would take into account the key findings of the review. There has been unquestioning implementation of mentoring programs. The review creates the rationale and need for providing formal mentoring programs with internal flexibility, such that mentoring strategies are tailored in collaboration with targeted Indigenous Australian communities to address their needs and preferences. Such tailoring is likely to be crucial for optimising acceptability and feasibility of program delivery as well as outcomes. Longer-term investments in evolving integrated models of mentoring with wrap-services (including families, communities, social services and other support systems) are germane to the development of effective mentoring practices for Indigenous populations in Australia. Key elements to consider for inclusion in such models are formal mentoring programs with internal flexibility; context-mapping; culturally-tailored programs; voluntary participation; mentor competencies (including both mentor training and cultural competence); regular one-on-one mentor contact; personal and social development; specialised skills and learning opportunities; long-term mentoring relationships; exit strategies; and celebrating Indigeneity within the program.

Methodologically, the review showed a predominance of descriptive publications in the field of Indigenous Australian mentoring across time. Descriptive research does not provide quality improvement in programs, nor does it provide evidence of how to improve programs or whether those programs were effective. There must be change in the patterns of research if the field is to advance. There is a real need to evaluate programs particularly in terms of outcomes and, given there were no economic evaluations, costs. Commitments to improving funding structures and attitudes toward research must change to improve mentoring outcomes across a range of social determinants of health for Indigenous Australians. While this review undertook to study mentoring for Indigenous Australians only, it is possible for the findings to be extrapolated to other similar structures, settings and populations.

### Endnotes

^a^Empowerment is defined in this paper as: “a social action process by which individuals, communities, and organizations gain mastery over their lives in the context of changing their social and political environment to improve equity and quality of life” [46].

^b^It should be noted that the three AIME [34–36] reports did cite specific measurable impacts but did not meet criteria to be classified as original research.
